# Editorial: Advances and Updates in Diffuse Cystic Lung Diseases

**DOI:** 10.3389/fmed.2021.691688

**Published:** 2021-06-07

**Authors:** Bruno Guedes Baldi, Souheil El-Chemaly, Kai-Feng Xu

**Affiliations:** ^1^Pulmonary Division, Heart Institute (InCor), University of Sào Paulo Medical School, Sào Paulo, Brazil; ^2^Division of Pulmonary and Critical Care Medicine, Brigham's and Women's Hospital, Harvard Medical School, Boston, MA, United States; ^3^State Key Laboratory of Complex Severe and Rare Diseases, Department of Pulmonary and Critical Care Medicine, Peking Union Medical College Hospital, Chinese Academy of Medical Sciences, Beijing, China

**Keywords:** diagnostic imaging, genetics, diffuse cystic lung disease, pathogenesis, treatment

This Frontiers Research Topic aimed to update relevant information, to publish advances and to determine future research directions regarding diffuse cystic lung diseases (DCLDs), such as lymphangioleiomyomatosis (LAM), pulmonary Langerhans cell histiocytosis (PLCH), and Birt-Hogg-Dubé (BHD) syndrome.

Diffuse cystic lung diseases are a variety group of diseases characterized by the presence of multiple cysts in more than one lung lobe, usually bilateral (1). Chest high-resolution computed tomography (HRCT) is the main tool and a multidisciplinary discussion is desirable in the approach to DCLDs. The main etiologies of DCLDs include LAM, PLCH, and other smoking-related diseases, BHD syndrome, lymphocytic intertitial pneumonia, pulmonary amiloidosis, bronchiolitis, metastatic neoplasms, and light-chain deposition disease, etc. (2, 3). New disorders are continuously included in the differential diagnosis of DCLDs, and based on the fact that management and prognosis vary according to the etiology, it is important to try to narrow such differential and, if possible, to confirm the cause, which might require a lung biopsy (1, 4).

Several advances were obtained in the understanding of DCLDs in the last decades, especially in the patogenesis, genetics, and in the diagnostic approach of LAM and PLCH, and, additionally, in the treatment of LAM (5–7). Despite improvements in such issues, there is still no curative treatment for LAM and PLCH (5, 7). Furthermore, the pathophysiology and natural history of several DCLDs are still not fully understood.

Therefore, new studies addressing DCLDs are desired to expand knowledge in the area, and to improve the management of patients. Five studies were published in this Research Topic, two about LAM, two focusing on BHD syndrome and a review about PLCH, highlighting diverse topics.

Previous studies demonstrated that serum matrix metalloproteinases (MMP) −2 and −9 were higher in LAM compared to healthy controls (8, 9). However, doxycycline, a MMP inhibitor, had no effects on pulmonary function decline in a clinical trial in LAM (10). Serum vascular endothelial growth factor (VEGF) -D is a useful biomarker included in the diagnostic guidelines of LAM (5). Previous studies demonstrated that serum VEGF-D was greater in patients with LAM than in healthy controls and in those with other cystic lung diseases (11, 12). Terraneo et al. assessed the role of MMP-2, MMP-7, VEGF-C, and VEGF-D in women with LAM and TSC. The authors found that MMP-2 was greater in LAM compared to controls and TSC patients, and that MMP-7 was higher in TSC-LAM patients. The authors reinforced the importance of VEGF-D in the diagnosis of LAM, and demonstrated that VEGF-C seems to have no role in the diagnosis of LAM and TSC. The authors concluded that MMP-2 and MMP-7 are promising biomarkers in LAM, although further studies with larger populations and with other DCLDs are required to confirm their findings.

Song et al. provided a comprehensive review of the pathogenesis of LAM and discussed the role of the mTOR signaling pathway in cell growth and proliferation. Although the authors highlighted the role of mTOR inhibitors in attenuating the lung function decline in LAM, they reinforced that such drugs are not a curative therapy (6). Furthermore, other potential therapeutic targets for LAM were presented in this review, such as inhibition of autophagy, inhibition of receptor tyrosine kinases, including VEGF receptors, hormonal blockade, urokinase-type plasminogen activator, blockade of the mTORC2 pathway, and DNA damage checkpoint (13, 14). This review supports the idea that in the future the treatment of LAM will be based on the combination of drugs, acting on different pathogenic pathways.

Zong et al. presented a novel heterozygous variant (c.912delT/p.E305KfsX18) in exon 9 of the folliculin gene, a tumor supressor, in seven patients with BHD syndrome from a Chinese family. All patients presented with bilateral pulmonary cysts on HRCT, three had primary spontaneous pneumothorax (PSP), and none had cutaneous or renal lesions. This case series strengthens the importance of screening for folliculin gene variants in patients with pulmonary cysts and PSP, and of trying to establish a correlation between genotype and phenotypic presentation, although it is not always clear in genetic disorders.

Muller et al. performed a meta-analysis of published epidemiological data using Bayes equation to determine for the first time the prevalence of BHD syndrome and, additionally, information regarding PSP. The main findings of this study were as follows: (1) The prevalence of BHD in the general population was 1.86 (95% confidence interval: 1.16–3.00) per million; (2) The probability of having BHD syndrome among patients with apparent PSP was 9%; (3) The incidence rate of PSP in the general population was 8.69 (95% confidence interval: 6.58–11.46) per 100,000 person-years; (4) The prevalence of PSP was 0.77 (95% confidence interval: 0.52–1.02) per 100,000; (5) The overall probability of PSP in BHD syndrome was 43%. Therefore, this study reinforces that BHD syndrome is rare and has a high probability of developing PSP, and the recommendation to screen for BHD syndrome in patients with PSP.

Diverse topics about PLCH are discussed in this broad narrative review by Radzikowska. The author presented updated issues about PLCH, emphasizing definition, classification, epidemiology, pathogenesis, genetic features, clinical manifestations, radiological and histological presentations, and diagnostic approach. Moreover, potential therapeutic modalities are highlighted in such review, including smoking cessation, corticosteroids, chemotherapy and target drugs. Future perspectives in PLCH include to expand the understanding of the pathogenesis, the identification of biomarkers to support diagnosis, prognosis and therapeutic response, to define efficacy and enhance experience with target drugs, and to identify curative treatments.

In summary, the manuscripts included in this Research Topic highlighted diverse issues and added relevant data to improve knowledge in the area of DCLDs. Although several advances were obtained in the genetic characterization, pathogenesis and diagnostic approach regarding DCLDs in recent years, there is still a need to identify novel targets and therapeutic modalities with curative results, especially in LAM and PLCH.

## Author Contributions

All authors wrote, reviewed, and approved the final version of the manuscript.

## Conflict of Interest

The authors declare that the research was conducted in the absence of any commercial or financial relationships that could be construed as a potential conflict of interest.

## References

[1] Baldi BG Carvalho CRR Dias OM Marchiori E Hochhegger B. Diffuse cystic lung diseases: differential diagnosis. J Bras Pneumol. (2017) 43:140–9. 10.1590/s1806-37562016000000341 28538782 PMC5474378

[2] Gupta N Vassallo R Wikenheiser-Brokamp KA McCormack FX. Diffuse cystic lung disease. Part I. Am J Respir Crit Care Med. (2015) 191:1354–66. 10.1164/rccm.201411-2094CI 25906089 PMC5442966

[3] Gupta N Vassallo R Wikenheiser-Brokamp KA McCormack FX. Diffuse cystic lung disease. Part II. Am J Respir Crit Care Med. (2015) 192:17–29. 10.1164/rccm.201411-2096CI 25906201 PMC5447298

[4] Raoof S Bondalapati P Vydyula R Ryu JH Gupta N Raoof S. Cystic lung diseases: algorithmic approach. Chest. (2016) 150:945–65. 10.1016/j.chest.2016.04.026 27180915 PMC7534033

[5] McCormack FX Gupta N Finlay GR Young LR Taveira-DaSilva AM Glasgow CG. Official American thoracic society/Japanese respiratory society clinical practice guidelines: lymphangioleiomyomatosis diagnosis and management. Am J Respir Crit Care Med. (2016) 194:748–61. 10.1164/rccm.201607-1384ST 27628078 PMC5803656

[6] McCormack FX Inoue Y Moss J Singer LG Strange C Nakata K. Efficacy and safety of sirolimus in lymphangioleiomyomatosis. N Engl J Med. (2011) 364:1595–606. 10.1056/NEJMoa1100391 21410393 PMC3118601

[7] Shaw B Borchers M Zander D Gupta N. Pulmonary langerhans cell histiocytosis. Semin Respir Crit Care Med. (2020) 41:269–79. 10.1093/med/9780198746690.003.0425 32279297

[8] Pimenta SP Baldi BG Acencio MMP Kairalla RA Carvalho CRR. Doxycycline use in patients with lymphangioleiomyomatosis: safety and efficacy in metalloproteinase blockade. J Bras Pneumol. (2013) 37:424–30. 10.1590/S1806-37132011000400003 21881731

[9] Chang WYC Cane JL Blakey JD Kumaran M Pointon KS Johnson SR. Clinical utility of diagnostic guidelines and putative biomarkers in lymphangioleiomyomatosis. Respir Res. (2012) 13:34. 10.1186/1465-9921-13-34 22513045 PMC3431996

[10] Chang WYC Cane JL Kumaran M Lewis S Tattersfield AE Johnson SR. A 2-year randomised placebo-controlled trial of doxycycline for lymphangioleiomyomatosis. Eur Respir J. (2014) 43:1114–23. 10.1183/09031936.00167413 24311763

[11] Young LR Vandyke R Gulleman PM Inoue Y Brown KK Schmidt LS. Serum vascular endothelial growth factor-D prospectively distinguishes lymphangioleiomyomatosis from other diseases. Chest. (2010) 138:674–81. 10.1378/chest.10-0573 20382711 PMC2940071

[12] Xu K-F Zhang P Tian X Ma A Li X Zhou J. The role of vascular endothelial growth factor-D in diagnosis of lymphangioleiomyomatosis (LAM). Respir Med. (2013) 107:263–8. 10.1016/j.rmed.2012.10.006 23127572

[13] El-Chemaly S Taveira-Dasilva A Goldberg HJ Peters E Haughey M Bienfang D. Sirolimus and autophagy inhibition in lymphangioleiomyomatosis: results of a phase I clinical trial. Chest. (2017) 151:1302–10. 10.1016/j.chest.2017.01.033 28192114 PMC6026235

[14] Atochina-Vasserman EN Abramova E James ML Rue R Liu AY Ersumo NT. Pharmacological targeting of VEGFR signaling with axitinib inhibits Tsc2-null lesion growth in the mouse model of lymphangioleiomyomatosis. Am J Physiol Lung Cell Mol Physiol. (2015) 309:L1447–54. 10.1152/ajplung.00262.2015 26432869 PMC4683311

